# Comparison of the diversity and structure of the rhizosphere microbial community between the straight and twisted trunk types of *Pinus yunnanensis*

**DOI:** 10.3389/fmicb.2023.1066805

**Published:** 2023-02-23

**Authors:** Peiling Li, Dan Zong, Peihua Gan, Hailin Li, Zhiyang Wu, Fahong Li, Changlin Zhao, Laigeng Li, Chengzhong He

**Affiliations:** ^1^Key Laboratory for Forest Genetics and Tree Improvement and Propagation in Universities of Yunnan, Southwest Forestry University, Kunming, China; ^2^Key Laboratory of Biodiversity Conservation in Southwest China, State Forestry Administration, Southwest Forestry University, Kunming, China; ^3^Key Laboratory for Forest Resources Conservation and Utilization in the Southwest Mountains of China, Ministry of Education, Southwest Forestry University, Kunming, China; ^4^College of Biodiversity Conservation, Southwest Forestry University, Kunming, Yunnan, China; ^5^National Key Laboratory of Plant Molecular Genetics, CAS Center for Excellence in Molecular Plant Sciences, Institute of Plant Physiology and Ecology, Chinese Academy of Sciences, Shanghai, China

**Keywords:** *pinus yunnanensis*, trunk type, rhizosphere microorganism, microbial community, microbial diversity

## Abstract

**Background:**

*Pinus yunnanensis* is a major silvicultural species in Southwest China. Currently, large areas of twisted-trunk *Pinus yunnanensis* stands severely restrict its productivity. Different categories of rhizosphere microbes evolve alongside plants and environments and play an important role in the growth and ecological fitness of their host plant. However, the diversity and structure of the rhizosphere microbial communities between P. yunnanensis with two different trunk types—straight and twisted—remain unclear.

**Methods:**

We collected the rhizosphere soil of 5 trees with the straight and 5 trees with the twisted trunk type in each of three sites in Yunnan province. We assessed and compared the diversity and structure of the rhizosphere microbial communities between *P. yunnanensis* with two different trunk types by Illumina sequencing of 16S rRNA genes and internal transcribed spacer (ITS) regions.

**Results:**

The available phosphorus in soil differed significantly between *P. yunnanensis* with straight and twisted trunks. Available potassium had a significant effect on fungi. *Chloroflexi* dominated the rhizosphere soils of the straight trunk type, while *Proteobacteria* was predominant in the rhizosphere soils of the twisted trunk type. Trunk types significantly explained 6.79% of the variance in bacterial communities.

**Conclusion:**

This study revealed the composition and diversity of bacterial and fungal groups in the rhizosphere soil of *P. yunnanensis* with straight and twisted trunk types, providing proper microbial information for different plant phenotypes.

## Introduction

*Pinus yunnanensis* is an endemic tree species in Southwest China that makes up one of the principal subtropical coniferous forests (Gao et al., 2021). It is distributed in a variety of geological areas ranging from 23° to 30° N and 96° to 108° E and grows in a continuous distribution at elevations ranging from 700 to 3,000 m in the Yunnan–Guizhou region (Wang et al., 2013; Gao et al., 2021). *P. yunnanensis* plays an important role in forestry economic development and environmental regulation in China (Xu et al., 2016; Wang X. et al., 2021). The degradation of forest stands of *P. yunnanensis* is becoming increasingly serious, leading to a growing proportion of low-quality stands (e.g., twisted, stunted, bending) (Zhou et al., 2016), which severely restrict the utilization and development of *P. yunnanensis*. Nevertheless, to date, the cause for the formation of twisted trunk characteristics remains unclear.

Much attention has been given to the cause of trunk twisting. On the one hand, many researchers have suspected that the causes of trunk twists are the wind, Earth’s rotation, injuries, directional aspects, exposure, and the sun and moon (Kubler, 1991; Eklund and Säll, 2000) as well as soil nutrient status and other soil conditions (Copisarow, 1933; Harris, 1989). On the other hand, it has been reported that trunk twisting is under considerable genetic control, such as in *P. radiata* (Burdon and Low, 1992; Gapare et al., 2007), *Picea sitchensis* (Hansen and Roulund, 1997) and *Picea abies* (Silva et al., 2000). Regarding trunk twisting in *P. yunnanensis*, Chinese researchers also have different viewpoints. Chen and Lyu (1962) believed that the density was the basic factor for the occurrence and development of twisting and that the wind promoteed and strengthened twisting. Zhou (1974) thought that strong sunlight and periodic excessive moisture were the main causes. However, many studies have shown that the trunk type is genetically controlled (Cai et al., 2016). Combining investigation methods with traditional breeding, it was found that the twisted trunk phenotype of parents could be passed on to offspring (Chen et al., 1997). Some researchers hold the view that the formation of a large number of twisted trunks in *P. yunnanensis* is caused by the extensive selection logging of natural forests at early stages, the artificial negative selection of natural disasters and unclear germplasm sources for afforestation (Chen et al., 2014; Wang X. et al., 2021). Modern molecular biology methods also revealed that trunk twisting is mainly regulated by genetic factors (He, 1994; Zhou et al., 2016), but currently, researchers believe that the synergistic effect of environmental factors (biotic and abiotic) enhance the incidence of this twisted feature (Wu et al., 2022). Our previous transcriptome data from phloem showed that the differentially expressed genes (DEGs) between *P. yunnanensis* with the straight and twisted trunk types were mainly involved in the interaction between plants and microorganisms (not published).

Roots not only provide plants with mechanical support, water and nutrients (Molefe et al., 2021), but also exude an enormous range of potentially valuable compounds into the rhizosphere (Walker et al., 2003; Bais et al., 2006). Cumulative evidence suggests that these compounds play an invaluable role in determining the interactions between roots and, eventually, the dynamics of plant and soil communities (Bais et al., 2006; Broeckling et al., 2008; Chai and Schachtman, 2022). The rhizosphere encompasses the millimeters of soil surrounding a plant root that is home to an overwhelming number of microorganisms (Berendsen et al., 2012; Philippot et al., 2013). Recent studies have revealed that different species of plants, or even different plant traits of the same species, assemble different rhizosphere microbial communities in the same soil environment (Arafat et al., 2017; Sayer et al., 2017; Bickford et al., 2020). These complex plant-associated microbial communities are crucial for plants because they can affect plant growth, productivity, nutrients and immunity directly or indirectly (Van Wees et al., 2004; Egamberdieva et al., 2010; Erturk et al., 2010; Berendsen et al., 2012; Patel, 2018; Tahir et al., 2019; Gu et al., 2020; Song Q. et al., 2021), as well as affect host phenotypes and adaptability (Herrera Paredes et al., 2018). For instance, Finkel et al. (2020) found that rhizosphere microorganisms can promote root growth by affecting plant hormone levels in *Arabidopsis*. Additionally, Wagner et al. (2014) confirmed that rhizosphere microorganisms can affect flowering phenology and selection on flowering time. There is growing evidence of the ability of rhizosphere microorganisms to alter the root morphological structure and improve root functions, which in turn improves plant nutrient uptake and physiology (Vacheron et al., 2013; Castellano-Hinojosa et al., 2021). Furthermore, some rhizosphere microorganisms can also severely constrain plant growth and development. Some studies have demonstrated that the dysbiosis of the protective bacterial communities in rhizosphere soil promotes the incidence of disease while manifestingin phenotypes that differ from those of healthy plants (van der Heijden et al., 2008; Hu et al., 2018; Lee et al., 2020). In addition, the rhizosphere microbiota is a very important source of endophytic microorganisms (Chi et al., 2004; Vandana et al., 2021). Microorganisms have been observed to enter host plants through roots and colonize plants (Chi et al., 2005; Thomas and Reddy, 2013). It has been reported that among the various plant-associated microbiota, endophytic microorganisms exhibit the most intimate and specific association with host plants (Vasileva et al., 2019; Mishra et al., 2022). There is growing evidence that the endophytic microorganisms support plants against both biotic and abiotic stresses (Ludwig-Muller, 2015; Sugio et al., 2015). For example, they directly or indirectly promote plant growth by inhibiting the growth of plant pathogens, producing various secondary metabolites (Giménez et al., 2007; Kang et al., 2007; Balmer et al., 2013; Yan et al., 2019; Matsumoto et al., 2021; Shubhransu et al., 2021; Vandana et al., 2021) and impacting the host phenotype (Jousset et al., 2017; Hassani et al., 2018; Harrison et al., 2021). Thus, it is critical to study the diversity and function of rhizosphere microorganisms and their contribution to a healthy plant.

Plants survive and evolve in the presence of microorganisms, which could be pathogenic or symbiotic (Hartmann et al., 2014). The consensus reached is that plant performance and activities can be fully described and understood only when plants and closely related microflora are considered (Zilber-Rosenberg and Rosenberg, 2008). In other words, the plant is considered an organism harboring microbial populations (Rosier et al., 2016). Previous studies on plant rhizosphere microbes have been mostly conducted on model plants and cash crops, such as *Arabidopsis thaliana* (Robbins et al., 2018), maize (Aira et al., 2010; Molefe et al., 2021), and wheat (Egamberdieva et al., 2008; Rossmann et al., 2020). Nevertheless, the diversity of the rhizosphere microbiota of *P. yunnanensis* remains unclear, including the differences in rhizosphere microbiota between the twisted and straight trunk types.

In the present study, to further understand the relationship between trunk type and microorganisms in *P. yunnanensis*, we hypothesized that the diversity and structure of the rhizosphere microbiota of *P. yunnanensis* with the twisted trunk type would differ significantly from that of the straight trunk type. Based on the above, our objectives were to (i) compare the diversity and structural characteristics of the rhizosphere microbial communities between the straight and twisted trunk types of *P. yunnanensis* base on high-throughput sequencing and statistical methods, and (ii) estimate the interactions of *P. yunnanensis* with rhizosphere microorganisms and soil properties and their effects on trunk type.

## Materials and methods

### Study sites and soil sample collection

We sampled rhizosphere and bulk soils of *P. yunnanensis* with two different trunk types at three representative growth sites, including 5 trees with straight and 5 trees with twisted trunk at each growth site. The growth sites (Supplementary Table 1) were located in the cities of Kunming, Chuxiong and Dali in Yunnan Province, within the longitudinal range from 99.85° to 103.23° E. The distance between the sites exceeds 200 km, with the farthest distance being between Kunming and Dali, reaching 760 km.

*P. yunnanensis* with the two different trunk types are intermixed in the forest stands under the same management (Supplementary Figure 1). After the removal of litter, we collected soil samples from the rhizosphere and bulk soil at a depth of 20 cm using a hoe. Rhizosphere soil, collected by using the shake-off method of Riley and Barber (1971), was immediately stored in liquid nitrogen, transported back to the laboratory, and stored at -80°C for microbial diversity analysis. The soil that had no plants growing nearby was considered bulk soil. The bulk soils collected were air-dried at ambient temperature for soil property measurement (Shi et al., 2011; Kamutando et al., 2017).

### Analysis of soil physical and chemical properties

Air-dried bulk soil samples were used to measure organic matter, pH, total and available nitrogen (N), phosphorus (P), and potassium (K), and catalase and sucrase activity. The soil organic matter was measured using the K_2_Cr_2_O_7_ (Walkley-Blach) method. The pH value was determined by the glass electrode method; total N by the Kjeldahl method; total P and available P by the molybdenum antimony resistance colorimetric method; total K by the NaOH melting-flaming luminosity method; available N by diffusion; and available K was determined in ammonium acetate (NH_4_OAc) extract by flame photometry (Zhang et al., 2014). Sucrase activity was measured using the 3,5-dinitrosalicylic acid colorimetric method, and catalase activity was determined by the KMnO_4_ titration method (Ge et al., 2018).

### DNA extraction and sequencing

Microbial DNA from rhizosphere soil samples was extracted using an MN NucleoSpin 96 Soi kit (Macherey-Nagel, Germany) following the manufacturer’s instructions. The concentration of the DNA extracted from the each sample was shown in Supplementary Table 2. The V3–V4 hypervariable regions of the 16S rRNA gene were amplified for each sample using barcoded universal primers 338F (5′-ACTCCTACGGGAGGCAGCA-3′)/806R (5′-GGACTACHVGGGTWTCTAAT-3′) (Guo et al., 2018). The ITS1F (5′-CTTGGTCATTTAGAGGAAGTAA-3′)/ITS2 (5′GCTCGTTCTTCATCGATGC-3′) primer pair was used to amplify the fungal internal transcribed spacer (ITS1) gene (Gardes and Bruns, 1993; Li et al., 2020). All high-throughput sequencing analyses of bacterial and fungal genes were performed based on the Illumina HiSeq 2500 platform (2 × 250 paired ends) at Biomarker Technologies Corporation (Beijing, China).

The raw data were quality-filtered using Trimmomatic (version 0.33) (Bolger et al., 2014), and the primer sequences were identified and removed by Cutadapt (version 1.9.1) (Martin, 2011). To obtain high-quality sequences, splicing of double-ended reads was performed using USEARCH (version 10), and chimeras were removed from the operational taxonomic unit (OTU) table using UCHIME (version 8.1) (Edgar et al., 2011; Edgar, 2013). The OTUs of bacteria and fungi were clustered at the 97% nucleotide identity threshold using USEARCH (version 10.0) (Edgar, 2013) with the GreenGenes Database (version 13.5) (DeSantis et al., 2006) and the Unite Database (version 8.0) (Kõljalg et al., 2005) as the reference. OTUs were filtered using 0.005% of all sequence numbers as a threshold (Bokulich et al., 2013).

### Statistical analysis

The differences in the microbial communities between the two groups were analyzed using linear discriminant analysis (LDA) effect size (LEfSe) based on taxonomic composition at different classification levels.

## Results

### Soil properties and their correlations with microbial communities in different trunk types

The soil physical and chemical properties of the two different trunk types of *P. yunnanensis* were determined. As shown in Table 1, compared with the soil from the twisted trunk group, the soil from the straight trunk group had a significantly lower available P content (*P* = 0.02).

**TABLE 1:** The physical and chemical properties of soil in the sampling fields of twisted and straight trunk *P. yunnanensis.*

Soil physical-chemical property	S	T
SOM (g**⋅**kg^–1^)	14.67 @ 6.00	16.02 @ 7.00
pH	5.91 @ 0.27	5.6 @ 0.52
Total N (g**⋅**kg^–1^)	74.89 @ 37.69	64.32 @ 10.91
Total P (g**⋅**kg^–1^)	0.98 @ 0.01	0.99 @ 0.01
Total K (g**⋅**kg^–1^)	2.56 @ 1.18	2.50 @ 1.08
Available N (mg**⋅**kg^–1^)	52 @ 25.48	47.35 @ 10.98
Available P (mg**⋅**kg^–1^)*	5.47 @ 2.44	11.02 @ 5.36
Available K (mg**⋅**kg^–1^)	112.00 @ 52.70	94.78 @ 69.15
Sucrase activity (mg**⋅**g^–1^)	0.14 @ 0.09	0.12 @ 0.08
Catalase activity (mg**⋅**g^–1^)	1.02 @ 0.39	1.05 @ 0.44

SOM, soil organic matter; N, nitrogen; P, phosphorus; K, potassium; S, straight trunk type of *P. yunnanensis*; T, twisted trunk type of *P. yunnanensis*. *P < 0.05.

The impact of the soil physical and chemical properties on bacterial and fungal community variation was determined by Mantel tests (Table 2). The results showed that available K was correlated with fungal communities (*P* = 0.01).

**TABLE 2:** Mantel tests of the correlation between the content of soil properties and the relative abundance of microbia at the genus level.

Factor	Bacteria		Fungi	
	*R* ^2^	*p*	*R* ^2^	*p*
SOM	0.04	0.33	0.15	0.08
pH	-0.03	0.55	-0.07	0.75
Total N	0.01	0.41	-0.17	0.90
Total P	NA	NA	NA	NA
Total K	0.03	0.34	0.00	0.48
Available N	-0.14	0.82	-0.06	0.65
Available P	0.02	0.37	0.03	0.40
Available K	-0.08	0.72	0.27	0.01
Sucrase activity	-0.25	0.98	0.13	0.14
Catalase activity	0.02	0.39	0.07	0.27

SOM, soil organic matter; N, nitrogen; P, phosphorus; K, potassium.

### Rhizosphere microbial diversity and differences between trunk types

**FIGURE 1:**
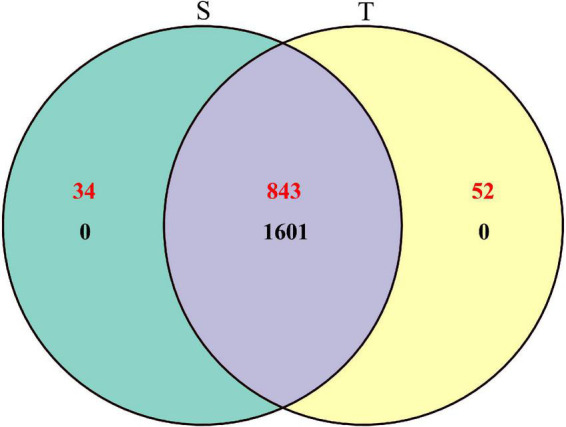
Venn diagram of specific and shared bacterial (black) and fungal (red) OTUs in straight and twisted trunk *P. yunnanensis*. S, straight trunk type of *P. yunnanensis*; T, twisted trunk type of *P. yunnanensis.*

To explore the rhizosphere soil bacterial and fungal communities of *P. yunnanensis* with two different trunk types, high-throughput sequencing of 16S rRNA and internal transcribed spacer (ITS) regions was performed for microbiomes from 30 rhizosphere samples. In total, we obtained 2,395,106 high-quality paired reads for bacteria and 2,472,255 paired reads for fungi, accounting for 96.40 and 98.04% of their raw tags, respectively (Supplementary Table 3). The composition and relative abundance for each taxon were obtained based on the OTU classification results. At the cut-off of 97% similarity (Edgar, 2013), the rhizosphere soil microbial communities of *P. yunnanensis* consisted of 1, 601 bacterial OTUs and 929 fungal OTUs. Specifically, 1,061 bacterial and 877 fungal OTUs were identified in straight-trunk *P. yunnanensis*, while 1,061 bacterial OTUs and 895 fungal OTUs were identified in twisted-trunk *P. yunnanensis* (Figure 1).

**FIGURE 2:**
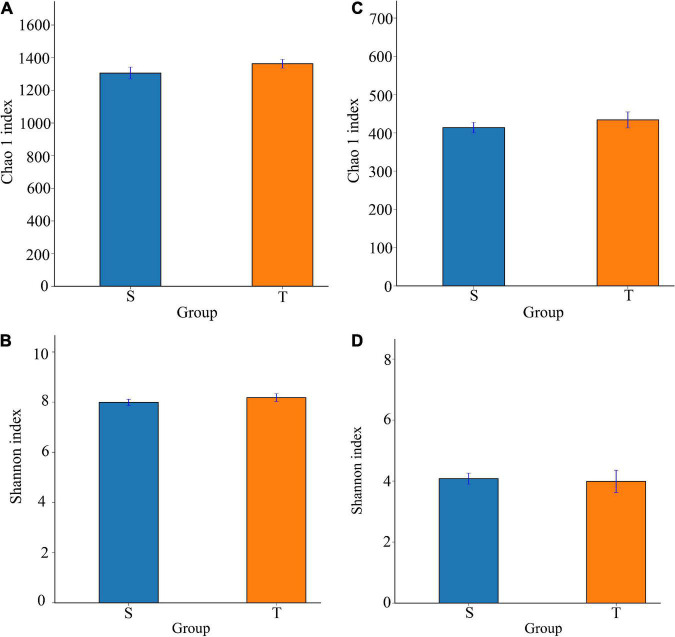
The Chao 1 (species richness), and Shannon (species diversity) indexes of bacterial and fungal communities for the straight and twisted trunk *P. yunnanensis*. Panels **(A,B)** represent Chao 1, and Shannon indexes of bacteria, respectively. Panels **(C,D)** represent Chao 1, and Shannon indexes of fungi, respectively. S, straight trunk type of *P. yunnanensis*; T, twisted trunk type of *P. yunnanensis.*

According to the Chao1 and Shannon-Wiener indices, we did not observe a significant difference in alpha diversity between the two trunk types (Figure 2). Moreover, the PERMANOVA results suggested that trunk type explained 6.79% of the variance in bacterial communities (*P* = 0.02) (Table 3).

**TABLE 3:** PERMANOVAs of the influence of trunk types on the microbial communities.

Factor	Bacterial community			Fungal community		
	F. Model	*R* ^2^	*p*	F. Model	*R* ^2^	*p*
Trunk types (S vs T)	2.04073	0.06793	0.022	1.10233	0.03788	0.136

S, straight trunk type of *P. yunnanensis*; T, twisted trunk type of *P. yunnanensis*.

### Taxonomic composition of bacterial communities

**FIGURE 3:**
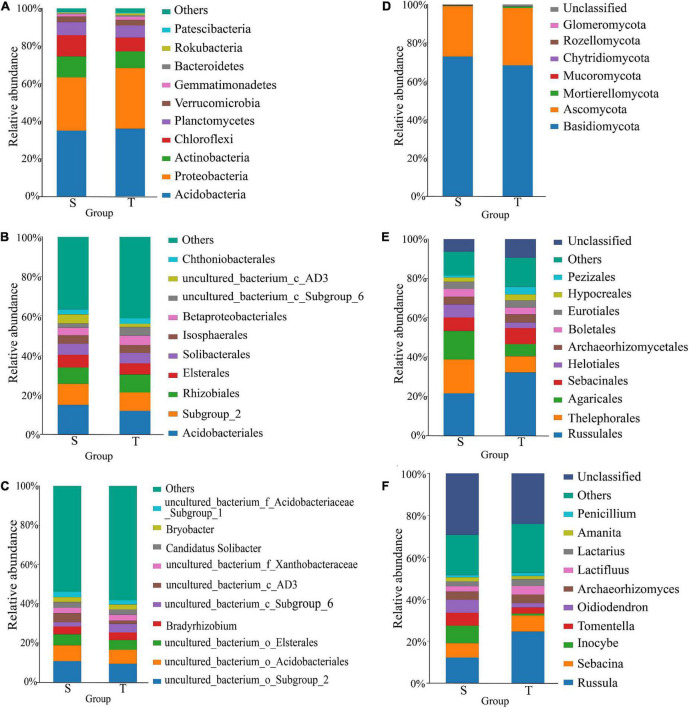
Taxonomic composition and structure of bacterial and fungi communities at phylum, order and genus levels. Panels **(A–C)** represent phylum, order and genus level of bacteria, respectively. Panels **(D–F)** represent phylum, order and genus level of fungi, respectively. S, straight trunk type of *P. yunnanensis*; T, twisted trunk type of *P. yunnanensis.*

We assessed the taxonomic composition of straight- and twisted-trunk *P. yunnanensis* rhizosphere microbial communities at different classification levels, including the phylum, order and genus levels. The results are shown in Figure 3. At the phylum level, there were 24 different bacterial phyla in both the straight and twisted groups. Among them, the relative abundances of *Proteobacteria* in the rhizosphere microbial communities of twisted-trunk *P. yunnanensi* were higher than those of straight-trunk *P. yunnanensis* (*P* = 0.02, Supplementary Figure 2). Furthermore, the relative abundance of *Chloroflexi* in twisted-trunk *P. yunnanensis* was significantly lower than that in straight-trunk *P. yunnanensis* (*P* = 0.04, Supplementary Figure 2). The same 142 rhizosphere bacterial orders were obtained in both the straight and twisted trunk groups. In the twisted trunk group, the relative abundance of *Pyrinomonadales* was 5.6 times higher than that in the straight trunk group, but the difference was not significant. The relative abundance of *uncultured_bacterium_c_Subgroup_6* was significantly higher in the twisted trunk group (*P* = 0.01, Supplementary Figure 2). At the genus level (Figure 3C), the bacterial community structure appeared more stable across the different compartments. Each of the two groups had 316 identical genera. *Subgroup_2* (10.76 and 9.07%, respectively), *Acidobacteriales* (8.09 and 7.18%, respectively), and *Elsterales* (5.52 and 4.87%, respectively) were the most abundant taxonomically known genera between the straight and twisted trunk groups. The relative abundances of *Acidobacteriales* and *uncultured_bacterium_c_AD3* were significantly higher in the straight trunk group (*P* = 0.03, Supplementary Figure 2). In addition, *Bradyrhizobium* was one of the most enriched bacterial genera in the rhizosphere microbial community of straight- and twisted-trunk *P. yunnanensis*.

### Taxonomic composition of fungal communities

We assessed the taxonomic structure of rhizosphere fungal communities at different classification levels by the same analysis method mentioned above. The composition of fungal communities comprised 7 different phyla, and 2 of them had a relative abundance greater than 1%, including *Basidiomycota* (72.96 and 68.35%, respectively) and *Ascomycota* (26.12 and 29.76%, respectively), with an overall relative abundance higher than 98% in the straight and twisted trunk groups (Figure 3D). The dominant fungal phyla in rhizosphere soil showed no clear difference between the straight and twisted trunk types of *P. yunnanensis*, as we estimated. In addition, there were 12 orders with an average relative abundance of more than 1% in the straight trunk group. The relative abundances of *Thelephorales, Agaricales* and *Helotiales* in the straight trunk group were more than twice as high as those in the twisted trunk group (Figure 3E). Nevertheless, such a difference was not significant due to the larger dispersion value. We identified a total of 222 and 223 fungal genera in the straight and twisted trunk groups, respectively. The results demonstrated that *Inocybe* (7.55 and 0.84%, respectively) was one of the genera with the greatest relative abundance variation between the straight and twisted trunk groups (Figure 3F). Furthermore, in the twisted trunk group, the relative abundances of both *Tylopilus* (1.64%) and *Hymenogaster* (1.36%) were greater than 1%. In contrast, in the straight trunk group, their relative abundances were less than 0.01%. The relative abundances of plant pathogens such as *Penicillium* and *Fusicolla* were higher in the twisted trunk group. At the species level, the relative abundance of *Penicillium nodositatum* was significantly higher in the twisted trunk group (*P* = 0.03, Supplementary Figure 2).

### Identification of microbial biomarkers for distinguishing different trunk types

**FIGURE 4:**
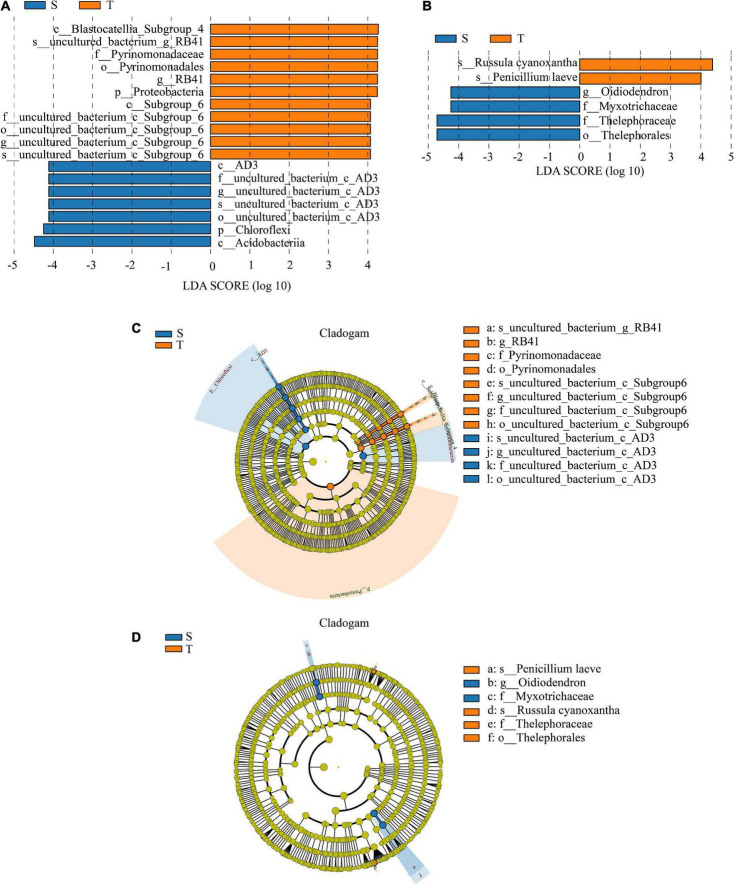
Histogram of LDA value distribution and evolutionary branch graph of LEfSe analysis. **(A)** The microbial biomarkers between different trunk shapes of *P. yunnanensis* in bacteria. **(B)** The microbial biomarkers between different trunk shape *P. yunnanensis* for fungi. **(C)** The different taxon between different trunk shape *P. yunnanensis* in bacteria. **(D)** The different taxon between different trunk shape *P. yunnanensis* in fungi. The circles from inner to outer layers represent the taxonomic level from phylum to species. The dots on circles represents a term on the corresponding taxonomic level. The size of the dots indicates relative abundance. Coloring: Species with no significant difference are colored in yellow, orange stand for twisted trunk group, and blue for straight. Species with certain color mean the abundance of this species is the highest in the corresponding group, which helps to visualize the most important microbial communities in each group. The lowercase p, c, o, f, g, and s in front of the symbol “_” represent the phylum, class, order, family, genus and species, respectively. S, straight trunk type of *P. yunnanensis*; T, twisted trunk type of *P. yunnanensis.*

To explore the indicator bacterial and fungal communities in rhizosphere soil of straight- and twisted-trunk *P. yunnanensis*, we conducted LEfSe analysis to identify biomarkers for each group based on the taxonomic composition of rhizosphere microbial communities with logarithmic LDA score > 4.0. The results showed that there were 18 distinctly abundant taxa in the two groups (Figures 4A, C). Specifically, 7 were differentially abundant in straight-trunk *P. yunnanensis*, including *Chloroflexi* and *Actinobacteria*. There were 11 taxa in the twisted-trunk, including *Pyrinomonadales* and *Proteobacteria*.

The LEfSe analysis of the fungal communities from the two trunk groups showed that 6 abundant fungal taxa presented significant differences, including *Penicillium laeve, Oidiodendron, Myxotrichaccac, Russula cyanoxantha, Thelephoraceae* and *Thelephorales*. *Myxotrichaceae* and *Thelephoraceae* were differentially abundant between the straight trunk and twisted trunk groups. The species *Penicillium laeve* and *Russula cyanoxantha* were more abundant in the twisted trunk group (Figures 4B, D).

## Discussion

To understand the soil conditions of straight and twisted trunk-type plants, we tested the physical and chemical properties of the soil around the roots. The results revealed that the available P content was significantly higher (*P* = 0.02) in the soil of twisted- than straight-trunk *P. yunnanensis*. Other physical and chemical properties were non-significantly different between the different trunk types of *P. yunnanensis*. The content of phosphorus and other nutrients in the soil represents the potential fertility of the soil (Song Q. et al., 2021). Soil fertility affects plant growth and the survival of microbes (Wang et al., 2020). For example, adequate phosphorus fertilization enhanced the seedlings growth and nutrient content of *P. massoniana* (Chen et al., 2022), and low fertility often tends to affect plant root structure and morphology to increase access to limiting nutrient resources (Zadworny et al., 2017). Kumar and Garkoti (2022) also reported that soil nutrients can influence the rhizosphere effect, which affects the transfer of energy and nutrients from the soil to plant roots. Furthermore, we found that available K was correlated with fungal communities (*P* = 0.01). As the environment for microbial life in the rhizosphere, soil properties influence the physiology and growth of soil microbial communities (Molefe et al., 2021). In addition, the associated soil nutrients (e.g., amount of C, N, and K) can change under biotic and abiotic environmental disturbances (Bastida et al., 2006; Otlewska et al., 2020; Crandall et al., 2022). Potassium-solubilizing microorganisms, widely present in different soil environments, can be used as biofertilizers to make available K from minerals and rocks, ultimately influencing soil nutrients, crop growth and quality (Das and Pradhan, 2016). The differential microorganisms in the rhizosphere of *P. yunnanensis* may disturb the balance of plant available nutrients in the soil and eventually lead to differences in the available P of different trunk types of *P. yunnanensis.* In turn, this difference can affect microorganisms. Moreover, our study revealed that trunk type explained 6.79 and 3.79% of the variance in bacterial (*P* = 0.02) and fungal communities, respectively. Perhaps the variance in microbial communities was determined by *P. yunnanensis* secreting compounds that specifically stimulate or inhibit the members of the microbial community (Bais et al., 2006; Arafat et al., 2017; Vives-Peris et al., 2020; Volpiano et al., 2022). In summary, we considered the soil environment, *P. yunnanensis* and microorganisms to interact and influence each other. We inferred that the trunk types of *P. yunnanensis* were influenced by microorganisms and soil properties, and vice versa.

Previous studies reported that plants can shape and recruit microbes from soil microbial communities to type rhizosphere microbial communities (Liu et al., 2021; Song Y. et al., 2021). Based on 16S rRNA gene sequence data, the rhizosphere bacterial community diversity in the straight and twisted trunk groups of *P. yunnanensis* was highly similar, including 24 phyla, 66 classes, 142 orders, 206 families, 316 genera and 334 species. The two different trunk types contained a similar relative abundance of rhizosphere microbial communities, which were dominated by *Acidobacteria, Proteobacteria, Actinobacteria, Chloroflexi* and *Planctomycetes* in *P. yunnanensis*. *Acidobacteria* constitutes the most abundant phylum whose members dominate soil bacterial communities (Liu et al., 2016). Here, we can exclude the effect of sampling distance on this result because the sampling sites were not close together. *Acidobacteria*, significantly enriched in the straight trunk group, are resistant to oxidative stress through the production of carotenoids, which may offer a competitive advantage for themselves in soils (Pinto et al., 2021). Their dynamic roles in vital ecological processes, including regulation of biogeochemical cycles, decomposition of biopolymers, exopolysaccharide secretion, and plant growth promotion have been investigated (Kalam et al., 2020). Surveys of root microbiomes suggested that certain members of the lineages cited above may be consistently enriched in the plant root environment (Yeoh et al., 2017). Some of them, such as *Proteobacteria* and *Actinobacteria*, occur in many plants as the dominant phyla, such as *Arabidopsis thaliana* (Bulgarelli et al., 2012), barley (Bulgarelli et al., 2015), lettuce (Schreiter et al., 2014), oak (Uroz et al., 2010), ginseng (Ying et al., 2012; Wang H. et al., 2021), *Dendrobium* (Zuo et al., 2021), and wheat (Rossmann et al., 2020). Furthermore, *Bradyrhizobium* was one of the most enriched bacterial genera in this study. Multiple studies have confirmed that co-inoculation of *Bradyrhizobium* and Plant Growth Promoting Rhizobacteria (PGPR) is beneficial to plant growth, including nodule biomass, root biomass, and shoot biomass, and they can be applied together as biofertilizers for production of economically important plants (Htwe et al., 2019; Zeffa et al., 2020).

According to our results, the twisted trunk rhizosphere microbiome group had slightly higher fungal OTUs and alpha diversity than the straight trunk group. The twisted trunk group consistently presented slightly higher bacterial alpha diversity than the straight trunk group. However, the differences were not significant. On the basis of the taxonomic composition, we observed that the relative abundances of *Thelephorales, Agaricales, Helotiales* and *Oidiodendron* in the straight trunk group were more than twice as likely as those in the twisted trunk group. *Oidiodendron* alters the length and branching of pioneer and fibrous roots of blueberry cuttings (Baba et al., 2021). Additionally, the relative abundances of *Penicillium* and *Fusicolla* were higher in the twisted trunk group. Unclassified genera accounted for 26.94%, which perhaps deserves further study in the future.

In this study, the LefSe analysis showed that *Proteobacteria* and *Penicillium laeve* were the key taxa in the rhizosphere soil of twisted-trunk *P. yunnanensis*. A large number of microorganisms in *Proteobacteria* (Tsolis, 2002; Preston et al., 2005) and *Penicillium* (Yang et al., 2017) are considered to be plant pathogens. In contrast, *Acidobacteria* and *Oidiodendron* were the key taxa in the rhizosphere soil of straight-trunk *P. yunnanensis*. *Acidobacteria* were reported to contribute to the healthy growth of their host plants and increase the chlorophyll content (Yoneda et al., 2021). *Oidiodendron* has a significant effect on root morphology (Baba et al., 2021).

Research on the effects of microorganisms on plants is extensive, both in terms of plant species and microbial species (Jacoby et al., 2017; Fitzpatrick et al., 2018; Caruso, 2020). Regarding conifers, Timonin (1964) found that disinfecting seeds of *P. banksiana* and *P. glauca* caused a significant reduction in seedling emergence. It was only later that researchers discovered that the lack of certain microorganisms on the seed surface may negatively affect seed germination and be detrimental to plant growth and development (Cardoso et al., 2011). Subsequent studies have found that rhizosphere microorganisms influence conifers in every way (Garcìa et al., 2004; Heredia-Acuña et al., 2018). For example, they influence the root length via hormones (Bent et al., 2001), shoot height and dry mass via phosphate solubilization (Singh et al., 2008), and growth via synergy or antagonism (Rojas et al., 2001). These effects determine the complex and variable phenotype of the plant. The variability occurs not only between species but also within a species. Understanding this variability is of key importance to improve the target products (Pot et al., 2002). Twisting is a representation of textured spirals (Gapare et al., 2007) and helical growth (Nakamura and Hashimoto, 2020). As a model plant, twisting has been studied in depth in *Arabidopsis thaliana* (Okada and Shimura, 1990; Buschmann et al., 2004; Nakamura and Hashimoto, 2020). However, the trunk twisting characteristics of trees have been less studied due to the long growth cycle and slow phenotypic shift of trees. To the best of our knowledge, there is no relevant report on the relationship between trunk types and microorganisms in conifers (e.g., *P. yunnanensis*). Rhizosphere microorganisms are one of the sources of endophytic microorganisms to plants, representing one of the important effects of microorganisms on plants. In the present study, we reported the first comprehensive investigation of microbial communities in rhizosphere soils between straight- and twisted-trunk *P. yunnanensis*.

Although our understanding of the effects and mechanisms of microbial action on plants is growing, there is no doubt that it is limited. The practical use and routine application of microorganisms remains a challenge, and it may take many years before our understanding is adequate to ensure their successful application in different systems (Cardoso et al., 2011). In this study, we could not determine that rhizosphere microorganisms contribute directly to the trunk types of *P. yunnanensis*, but the results provide us with some useful information for future studies on the causes of twisted trunk formation. Next, perhaps we will be able to determine the relationship between rhizosphere and endophytic microorganisms, determine the effect of a particular microorganism and/or available P on *P. yunnanensis* growth and trunk type and further investigate the effects of interactions of *P. yunnanensis* with microbes and environments on trunk types.

## Conclusion

We provided a detailed and systematic understanding of the rhizosphere microbiome composition between straight and twisted trunk types of *P. yunnanensis*. Our high-throughput sequencing results demonstrated that the diversity and community composition of the two trunk types were similar. Further analysis showed that *Proteobacteria* and *Penicillium laeve* were the key taxa in the rhizosphere soil of twisted-trunk *P. yunnanensis*. In contrast, *Acidobacteria* and *Oidiodendron* were the key taxa in the rhizosphere soil of straight-trunk *P. yunnanensis.* Moreover, available potassium has a significant effect on fungi. Trunk type explained 6.79 and 3.79% of the variance in bacterial and fungal communities, respectively. Available phosphorus differed significantly between the two trunk types of *P. yunnanensis.* These findings significantly advance our fundamental understanding of the rhizosphere microorganisms of *P. yunnanensis* and of the microbial diversity of different plant phenotypes.

## Data availability statement

The data presented in this study are deposited in the NCBI repository, accession numbers are PRJNA892260 and PRJNA892749.

## Author contributions

PL, DZ, CH, and LL planned and designed the experiment. PL, DZ, PG, ZW, and FL prepared the materials. PL and PG collected and analyzed the data. PL and DZ wrote the first draft of the manuscript. HL and CZ commented on previous versions of the manuscript. All authors read and approved the final manuscript.
