# Supplementary material for: Comparison of the diversity and structure of the rhizosphere microbial community between the straight and twisted trunk types of Pinus yunnanensis
Source: Front Microbiol. 2023 Feb 23;14:1066805. doi: 10.3389/fmicb.2023.1066805 (PMC9995709; doi:10.3389/fmicb.2023.1066805)
Supplement: Supplementary file 1 [file Data_Sheet_1.pdf]

Supplementary Material

Supplementary Figures and Tables

1 Supplementary Figures

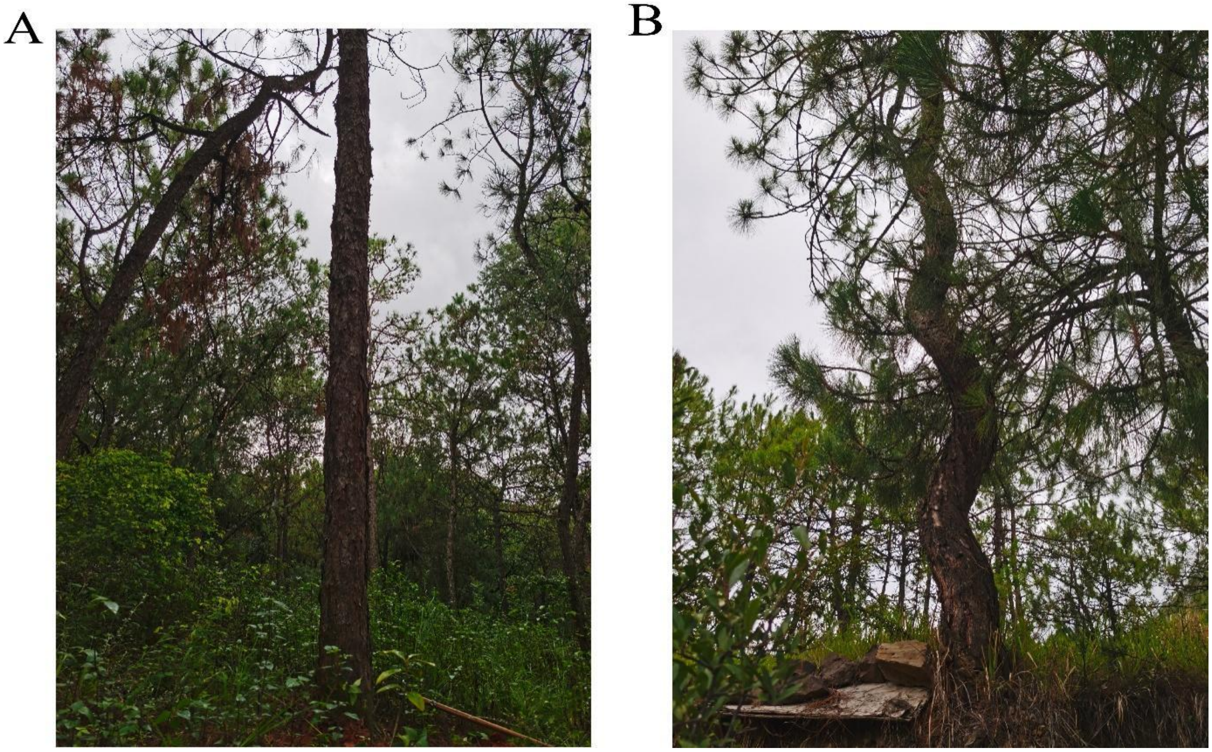

Supplementary Figure 1. Morphology character of straight (A) and twisted (B) trunk of *P. yunnanensis*.

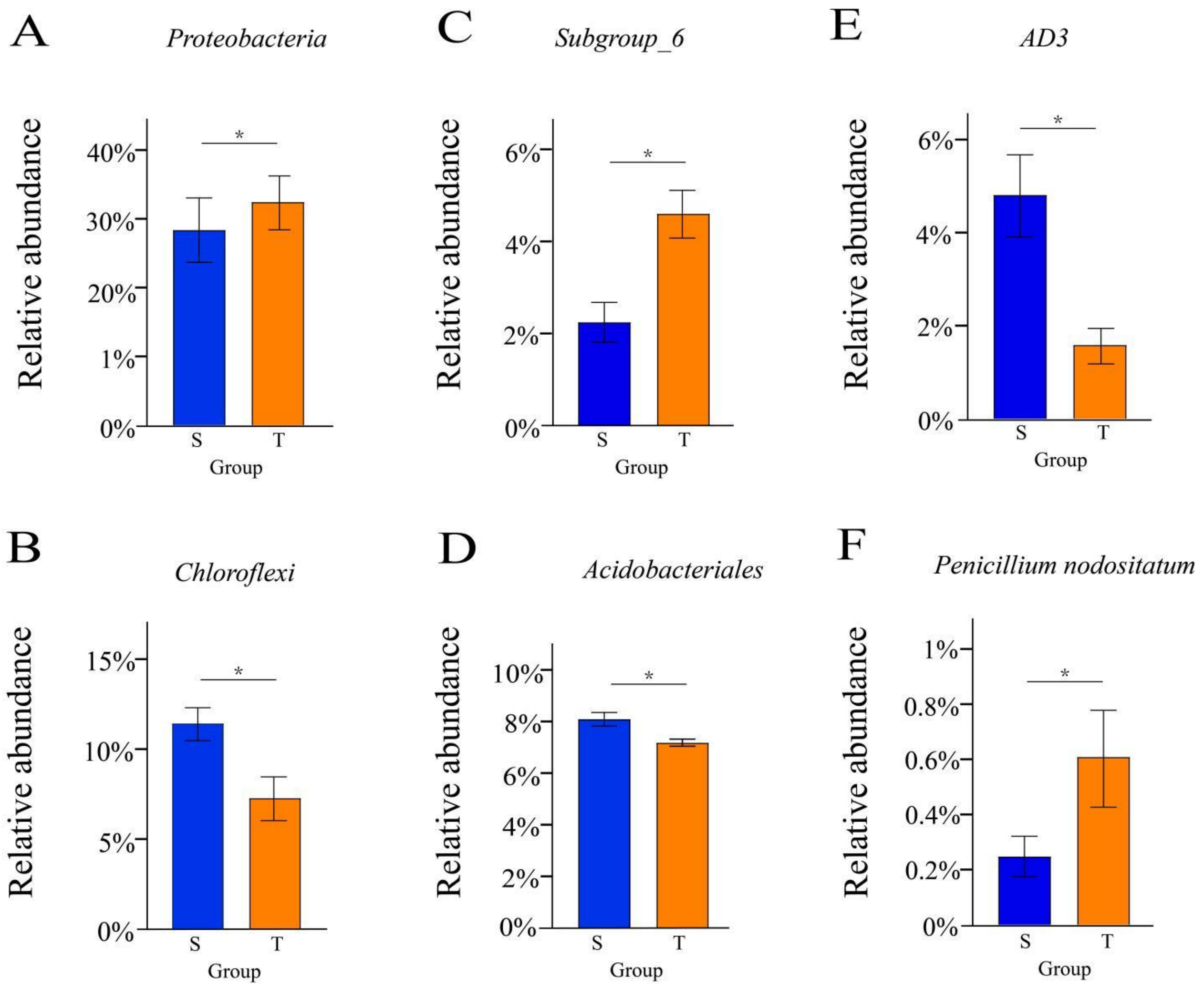

Supplementary Figure 2. Relative abundances of *Proteobacteria* (A), *Chloroflexi* (B), *uncultured_bacterium_c_Subgroup_6* (C), *Acidobacteriales* (D), *uncultured_bacterium_c_AD3* (E) and *Penicillium nodositatum* (F) in rhizosphere soils between straight and twisted trunk types of *P. yunnanensis.* S: Straight trunk type of *P. yunnanensis*; T: Twisted trunk type of *P. yunnanensis.* *$P < 0.05$.

2 Supplementary Tables

Supplementary Table 1. Sample information and geographic location.

Location	Latitude	Longitude	Altitude	Annual average temperature	Annual precipitation	NS	NT
Kunming	25.02° N	103.23° E	1660 m	17.4 ℃	958 mm	5	5
Chuxiong	26.12° N	101.78° E	1790 m	17.1 ℃	909 mm	5	5
Dali	26.43° N	99.85° E	2310 m	13.4 ℃	1043 mm	5	5

NS: The number of straight-trunk *P. yunnanensis*; NT: The number of twisted-trunk *P. yunnanensis*.

Supplementary Table 2. The concentration of the DNA extracted from the each sample.

Sample	Bacteria		Fungi	
	Concentration (ng/µL)	Conclusion	Concentration (ng/µL)	Conclusion
S-CX1	8.6	A	5.1	A
S-CX2	7.5	A	8.3	A
S-CX3	10.6	A	6.7	A
S-CX4	11.5	A	9.6	A
S-CX5	10.0	A	8.5	A
S-DL1	9.2	A	7.2	A
S-DL2	7.9	A	6.0	A
S-DL3	6.1	A	7.1	A

S-DL4	8.3	A	5.9	A
S-DL5	16.0	A	10.3	A
S-KM1	5.1	A	8.4	A
S-KM2	9.4	A	5.2	A
S-KM3	11.0	A	5.7	A
S-KM4	7.3	A	6.6	A
S-KM5	8.1	A	8.0	A
T-CX1	15.5	A	7.2	A
T-CX2	9.4	A	5.0	A
T-CX3	6.0	A	6.5	A
T-CX4	11.6	A	7.1	A
T-CX5	9.7	A	5.3	A
T-DL1	8.1	A	6.0	A
T-DL2	9.3	A	6.7	A
T-DL3	7.5	A	7.8	A
T-DL4	10.6	A	8.0	A
T-DL5	9.8	A	5.5	A
T-KM1	5.7	A	6.1	A
T-KM2	9.0	A	5.3	A
T-KM3	15.4	A	6	A

T-KM4	6.7	A	7.5	A
T-KM5	8.6	A	9.1	A

S: Straight trunk type of *P. yunnanensis*; T: Twisted trunk type of *P. yunnanensis*. CX: Chuxiong; DL: Dali; KM: Kunming. A: The quality of the sample is qualified and meets further research.

Supplementary Table 3. Number of processed sequencing reads and estimates for the diversity of each microbial community.

Sample	Bacteria			Fungi		
	Raw Tags	Effective Tags	Observed OTUs	Raw Tags	Effective Tags	Observed OTUs
S-CX1	155767	149770	790	411655	409148	335
S-CX2	154672	145632	1291	394708	393148	316
S-CX3	155769	147145	1152	303308	303201	296
S-CX4	154364	146010	1016	325072	324252	307
S-CX5	155370	144283	1129	313399	313317	384
S-DL1	154597	139235	1305	326980	325258	392
S-DL2	153626	143508	1296	362124	360981	375
S-DL3	155145	149600	1266	327903	327833	294
S-DL4	155139	141805	1292	329742	329231	349
S-DL5	155853	147096	1316	330646	330403	376

S-KM1	151148	142875	1244		376322	374493	315
S-KM2	151557	142311	1347		322108	321497	333
S-KM3	152397	129951	1115		367943	365992	453
S-KM4	154426	144557	1257		477079	471409	415
S-KM5	155188	140811	1087		310489	305885	359
T-CX1	153548	145005	1330		511889	497168	460
T-CX2	154794	145902	1341		324160	319920	465
T-CX3	155535	146143	1388		349681	349470	392
T-CX4	154293	128081	1343		391155	388803	560
T-CX5	154317	142585	1287		322277	317809	479
T-DL1	154451	144663	1298		304040	302142	354
T-DL2	155522	143466	1161		311009	310318	320
T-DL3	154004	147330	1196		335715	335615	323
T-DL4	154795	147294	1226		338472	338348	371
T-DL5	155364	145948	1335		328576	325987	325
T-KM1	151893	133506	810		354701	351016	267
T-KM2	151545	143433	1273		338726	338592	360

T-KM3	151702	141572	1258		348690	347012	285
T-KM4	151624	137808	1247		319965	318333	373
T-KM5	147186	135754	1303		494322	490826	301

S: Straight trunk type of *P. yunnanensis*; T: Twisted trunk type of *P. yunnanensis*. CX: Chuxiong; DL: Dali; KM: Kunming.
